# Comprehensive profiling of genomic invertons in defined gut microbial community reveals associations with intestinal colonization and surface adhesion

**DOI:** 10.1186/s40168-025-02052-7

**Published:** 2025-03-10

**Authors:** Xiaofan Jin, Alice G. Cheng, Rachael B. Chanin, Feiqiao B. Yu, Alejandra Dimas, Marissa Jasper, Allison Weakley, Jia Yan, Ami S. Bhatt, Katherine S. Pollard

**Affiliations:** 1 Gladstone Institutes, San Francisco, USA; 2 Department of Biomedical Engineering, University of Calgary, Calgary, Canada; 3 Department of Gastroenterology, Stanford School of Medicine, Stanford, USA; 4 Section of Gastroenterology, University of Chicago, Chicago, USA; 5 Division of Hematology, Stanford School of Medicine, Stanford, USA; 6 Arc Institute, Palo Alto, USA; 7 Sarafan ChEM-H Institute, Stanford University, Stanford, USA; 8 Chan Zuckerberg Biohub SF, San Francisco, USA; 9 Department of Genetics, Stanford University, Stanford, USA; 10 University of California San Francisco, San Francisco, USA

**Keywords:** Invertons, Phase variation, Gut microbiome, Adhesion, Colonization

## Abstract

**Background:**

Bacteria use invertible genetic elements known as invertons to generate heterogeneity among a population and adapt to new and changing environments. In human gut bacteria, invertons are often found near genes associated with cell surface modifications, suggesting key roles in modulating dynamic processes such as surface adhesion and intestinal colonization. However, comprehensive testing of this hypothesis across complex bacterial communities like the human gut microbiome remains challenging. Metagenomic sequencing holds promise for detecting inversions without isolation and culturing, but ambiguity in read alignment limits the accuracy of the resulting inverton predictions.

**Results:**

Here, we developed a customized bioinformatic workflow—PhaseFinderDC—to identify and track invertons in metagenomic data. Applying this method to a defined yet complex gut community (hCom2) across different growth environments over time using both in vitro and in vivo metagenomic samples, we detected invertons in most hCom2 strains. These include invertons whose orientation probabilities change over time and are statistically associated with environmental conditions. We used motif enrichment to identify putative inverton promoters and predict genes regulated by inverton flipping during intestinal colonization and surface adhesion. Analysis of inverton-proximal genes also revealed candidate invertases that may regulate flipping of specific invertons.

**Conclusions:**

Collectively, these findings suggest that surface adhesion and intestinal colonization in complex gut communities directly modulate inverton dynamics, offering new insights into the genetic mechanisms underlying these processes.

Video Abstract

**Supplementary information:**

The online version contains supplementary material available at 10.1186/s40168-025-02052-7.

## Introduction

Bacteria in the human gut microbiome exist in complex communities with fluctuating dynamics [1–4] and spatial structure [5–7]. Many gut-associated bacteria use phase variation, a mechanism of generating phenotypic diversity across individual cells of the same strain to adapt and persist in these changing environments [8–15]. One mechanism of phase variation is site-specific recombination of genomic DNA, often referred to as invertons [14]. Invertons are regions of DNA flanked by short (10–40 nucleotide) inverted repeats (IR) [14]. Invertase enzymes bind specifically to motifs within IR and mediate flipping of the intervening DNA sequence [16]. Inverton flipping rate is highly dependent on context; however, this enzyme mediated process is reversible and generally occurs more rapidly than other genomic alterations, such as nucleotide changes, insertions and deletions [17].

Invertons affect microbial phenotypes in multiple ways. Intergenic invertons, invertons contained entirely between coding regions, often regulate gene expression via flipping of inverton-embedded promoters turning transcription of adjacent regions on or off [8, 14]. Additionally, flipping of gene-intersecting invertons can lead to new protein isoforms or altered specificity [18, 19]. Of note, invertons are often found in high abundances in gut-associated bacteria [14] and are more likely to flip in complex versus uniform environments [18]. Invertons often regulate production of cell surface products such as exopolysaccharides, outer membrane proteins, and fimbriae [8–11, 13, 14], which are known to be associated with processes of gut colonization and surface adhesion [20–25] and can directly influence the human immune system [26].

Previous work has analyzed closely related genomes and next generation sequencing datasets of microbial cultures to identify a large number of invertons in gut-associated microbes [14, 18], while computational approaches have yielded additional inverton predictions based on comparative genomics [19] and deep learning [27]. However, the extent to which invertons modulate colonization and adhesion across a complex gut community remains unclear, as metagenomic approaches for comprehensive community-wide inverton profiling are limited by the general problem of sequence alignment ambiguity in communities with closely related strains [28].

We addressed these challenges by developing a bioinformatic workflow—PhaseFinderDC—to comprehensively profile invertons in defined communities of bacteria, extending and refining the original PhaseFinder algorithm [14]. We then applied this workflow on metagenomic samples of hCom2—a defined yet complex community of bacterial strains modeled after the human gut [29]—grown across multiple conditions. These included (i) isolate cultures of individual hCom2 strains [29, 30], (ii) fecal samples of gnotobiotic mice inoculated with hCom2 recovered from mice colonized over a total of 5 generations, and (iii) mixed cultures of hCom2 in various spatially structured in vitro environments [30] over a total of 6 passages. Our workflow successfully identified invertons in a majority of hCom2 strains and all eight represented phyla. For each identified inverton in each sample, we compared the proportion of sequencing reads supporting forward versus reverse inverton orientations. Using this approach, we identified a subset of “directionally biased” invertons whose orientation probabilities are significantly different between growth conditions (e.g., isolate culture versus in vivo mouse) and across timepoints (e.g., mouse generations or in vitro passages).

Categorizing the identified invertons into groups based on homology of their respective IR sequences, we applied motif enrichment analysis to identify motifs of IR and promoter sequences found in specific inverton groups. We identified gene families enriched in consistent orientations near invertons, highlighting cases where inverton-embedded promoters could potentially drive expression of “regulatable” downstream genes. We then used orientation of directionally biased invertons with regulatable genes to predict how surface adhesion and intestinal colonization dynamics are linked to expression of key bacterial genes, including surface-modifying genes such as those related to exopolysaccharide (EPS) biosynthesis in *Bacteroides*. Finally, we also highlight cases where specific invertase genes are enriched near specific inverton groups, potentially representing candidate invertases responsible for controlling inverton flipping. Together, these bioinformatic analyses provide a comprehensive community-wide characterization of inverton dynamics in a complex gut community, and point to key biological functions that modulate—and are modulated by—inverton-mediated phase variation.

## Results

### PhaseFinderDC detects invertons in defined communities with high precision

Using the original PhaseFinder algorithm [14] as a starting point, we developed PhaseFinderDC as a workflow to identify invertons in defined microbial communities for which reference genomes are available, with the ability to specifically discern invertons when closely related strains exist within the community. PhaseFinderDC was designed to take as input a concatenated reference genome database consisting of all strain genomes, and generate an alignment index by scanning this database for inverted repeats and compiling both forward and reverse orientation sequences for all inverted repeats (i.e., potential invertons), following the “locate” and “create” steps in the original PhaseFinder algorithm which located inverted repeats and created an alignment index, respectively. The alignment index created by the original PhaseFinder “locate” step consisted of forward and reverse orientation sequences—with flanking buffers—for all inverted repeats, but did not include the full intervening sequences of genomic regions between potential invertons. This alignment index (not the original input genome database) was then used in the PhaseFinder “ratio” step for bowtie-based alignment [31] of short-read sequence pairs. As a modification in the “create” step, PhaseFinderDC also included sequences corresponding to genomic regions between inverted repeats so that the final sequence index covered the entire concatenated genome database and not merely the inverted repeat regions. PhaseFinderDC then used bowtie2 in its “ratio” step to align short-read sequencing data to this index and quantify for each potential inverton the number of reads that align to the forward and reverse orientation references (Methods–Inverton detection using defined community sequencing data). Crucially, this comprehensive alignment index allowed filtering of alignments by mapping quality (MAPQ) score to identify multi-mapping reads. We designed PhaseFinderDC to discard all ambiguous multi-mapping reads (MAPQ < 30 by default) when counting forward (F) and reverse (R) supporting reads for each potential inverton. This conservative approach enabled PhaseFinderDC to confidently call invertons even when highly related strains were present in the defined microbial community, at the potential expense of missing invertons with a high degree of sequence homology.

**Fig. 1:**
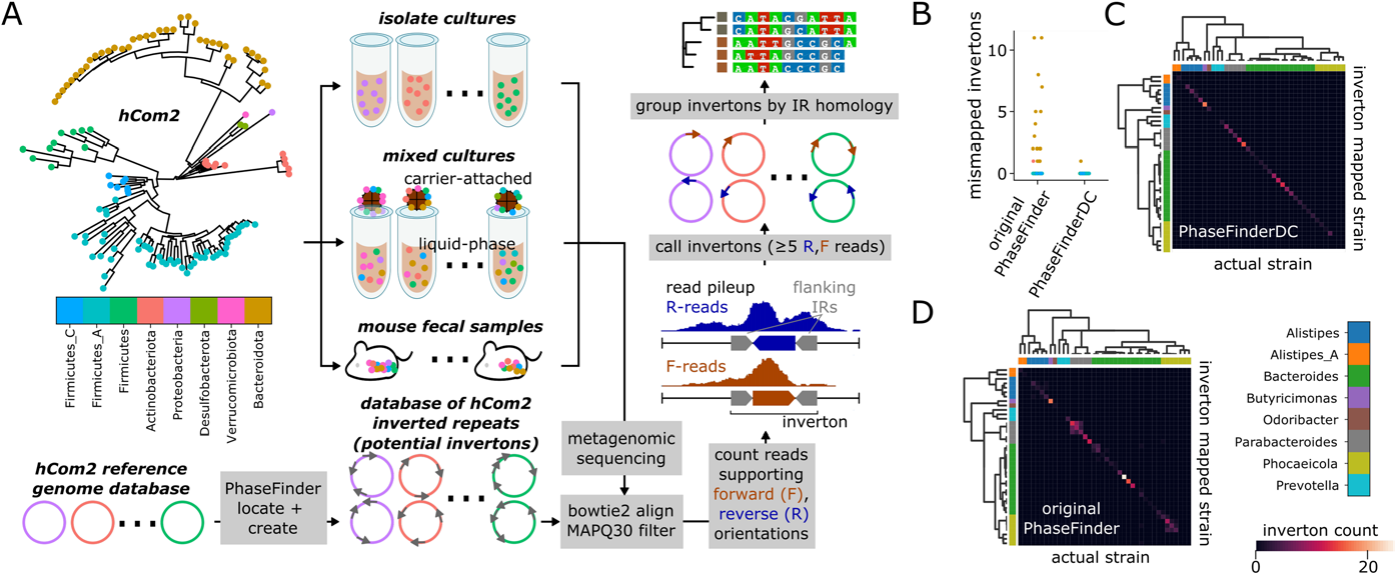
Customized workflow reliably detects invertons in metagenomes from defined communities. **A** Flowchart of customized workflow to detect and group invertons in hCom2 from metagenomic sequencing data. Briefly, PhaseFinderDC’s locate + create steps search the hCom2 reference genomes for inverted repeats (i.e., potential invertons) and create an augmented reference database including forward, reverse orientations for these repeats as well as intervening sequences. Shotgun metagenomic hCom2 short-read sequencing data is then mapped onto this database, and forward/reverse read counts are enumerated for each inverted repeat after MAPQ filtering for multi-mapping reads. Invertons are called if sufficient reverse/forward reads are detected, and then grouped by homology of their inverted repeat sequence. **B** Benchmarking of workflow precision based on pure isolate cultures—mismapped invertons refers to invertons called in one strain when using another strain’s sequencing library as input. **C** Heatmap of inverton counts among Bacteroides strains generated by PhaseFinderDC workflow, organized by actual strain (known based on isolate culture identity) and called inverton strain—off diagonal elements thus represent mismapped invertons. Strains are organized by phylogeny, margins correspond to genus. **D** Heatmap of inverton counts among Bacteroides strains generated by original PhaseFinder workflow, noting increase of off-diagonal (mismapped) calls between pairs of closely related strains

We applied PhaseFinderDC on metagenomic samples derived from the hCom2 defined microbial community [29], which included several instances of closely related strains (Methods–Inverton detection using defined community sequencing data). As all strains in hCom2 are known a priori, we used a customized genome database generated by concatenating all hCom2 strain genomes (Supplementary Table S1, Fig. 1A) as opposed to generic bacterial genome databases that would have included large numbers of strains beyond hCom2. To benchmark the precision of our updated PhaseFinder workflow, we used short-read sequencing data obtained from isolate cultures of each hCom2 strain (Supplementary Table S2) and quantified the number of reads that align to the forward and reverse orientation references for each potential inverton in hCom2. We called invertons based on read counts from these isolate cultures if support for forward and reverse orientations had at least 5 reads each (Methods–Inverton detection using defined community sequencing data). We quantified the proportion of invertons called in the correct versus incorrect strains, and found that our updated workflow produced 557 total calls, out of which 1 was incorrectly mapped (i.e., inverton called in a strain that was not the strain the sequencing data came from). This represented an improved precision of inverton calls compared to the original PhaseFinder workflow which called 69 incorrectly mapped invertons out of 439 total calls with the same input read libraries and reference metagenome database (Fig. 1B, Supplementary Table S3). This improvement was especially dramatic among strains from the phylum Bacteroidota, wherein hCom2 has several instances of closely related strains. Indeed, closer examination of invertons called in Bacteroidota confirmed that incorrectly mapped invertons tended to occur between closely related strains (Fig. 1C,D), consistent with the known pitfall of multi-mapping reads between closely related strains in metagenomic read alignment [28]. Together, these findings validated our updated workflow as a precise approach for detecting invertons in defined bacterial communities.

**Fig. 2:**
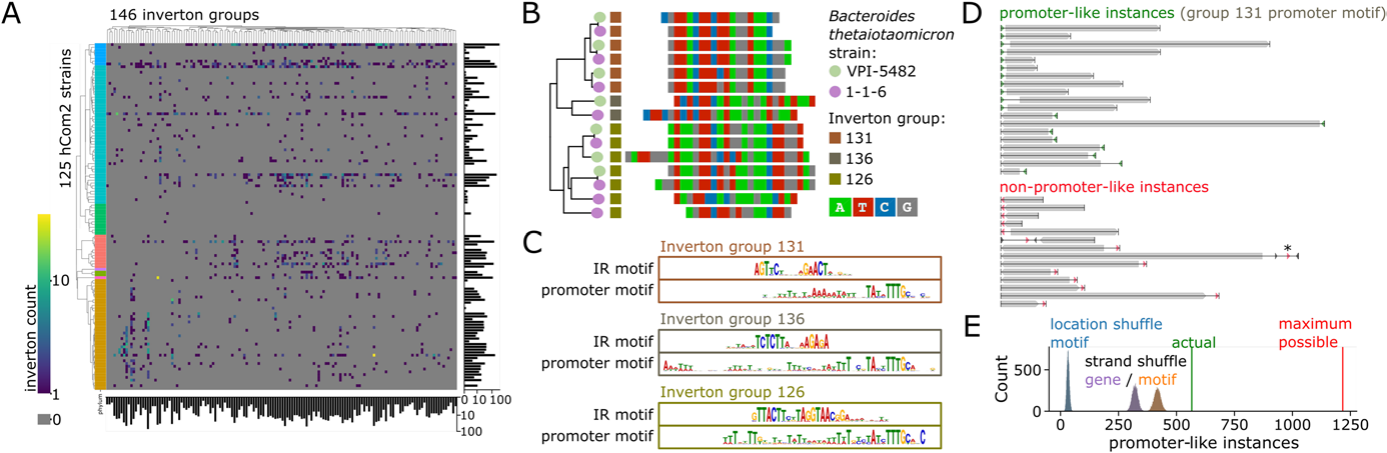
Enriched motifs detected in specific inverton groups grouped by IR sequence homology. **A** Log-heatmap of inverton counts by hCom2 strain and by inverton group. hCom2 strains organized by phylogeny, phylum colors as in Fig. 1A. **B** Example of tree-building/grouping of identified invertons, subset on a group of 15 invertons in 2 *Bacteroides thetaiotaomicron* strains that were classified into groups 126, 131, and 136. **C** Promoter and IR motifs detected as enriched in inverton groups 126, 131, and 136. Promoter motifs found in each of these groups highly resembled previously described Bacteroides promoter motif [14, 33]. **D** Instances of promoter-like motif from inverton group 131 (motif 131-2) found in *Bacteroides thetaiotaomicron* VPI-5482 genome tended to be oriented upstream of nearest gene (gray). Instances are colored in green if consistent with promoter-like orientation, red otherwise. Asterisk marks instance of inverton embedded promoter that if flipped would be consistent with promoter-like orientation. **E** Across all of hCom2, 566/1217 total instances of motif 131-2 are consistent with promoter-like orientation—more than would be expected by random chance, based on random shuffling of motif loci, permuting motif strand, or permuting gene strand (10,000 random samples tested for each case)

We applied PhaseFinderDC on a total of 636 sequencing samples from (i) isolate cultures of individual hCom2 strains, (ii) fecal samples of gnotobiotic mice inoculated with hCom2, and (iii) mixed in vitro cultures of hCom2 (Supplementary Table S2 for sequencing metadata). For each inverted repeat location, we summed the forward (F) and reverse (R) orientation read counts pooled across all 636 samples (Supplementary Table S4) and detected 1837 invertons (Methods–Inverton detection using defined community sequencing data, Supplementary Table S5) across 99/125 strains in all 8 phyla present in the hCom2 community (Fig. 2A, Supplementary Table S5). Inverton counts per genome exhibited a wide range from *Mitsuokella multacida* DSM-20544 with 181 detected invertons, to 26 genomes without any detected invertons. For the 5 phyla in hCom2 with more than 2 representative strains, we found higher occurances of invertons in Bacteroidota with median 8.5 invertons per genome (interquartile range IQR 4.75–16.25), Actinobacteriota with median 14 invertons per genome (IQR 7–23.75), and Firmicutes_C (primarily Negativicutes-like) with median 11 invertons per genome (IQR 2.5–54.5, note the NCBI phylum Firmicutes was split into Firmicutes_A, Firmicutes_C, and Firmicutes in GTDB) [32]. Lower inverton counts were observed in Firmicutes_A (primarily Clostridia-like) with median 3 invertons per genome (IQR 1–8.75) and Firmicutes (primarily Bacillus-like) with median 0 invertons per genome (IQR 0–0). Comparing the genomic loci of identified invertons against those of predicted gene coding sequences (CDS), we found that just over half the invertons—952/1837—intersect a CDS, while the rest were intergenic (Supplementary Table S5). Invertons in Bacteroidota are primarily intergenic, while the opposite is true for Firmicutes_C. Invertons in Actinobacteriota, Firmicutes_A, and Verrumicrobiota (with single representative strain *Akkermansia muciniphila*) ATCC-BAA-835 are roughly evenly split between intergenic and gene-intersecting (Supplementary Section S1).

Invertons exhibited a range of flipping frequencies: pooling reads across samples, F vs. R read counts in some invertons suggest nearly universal preference for a single orientation, while others were frequently found in both orientations. Estimating a "minor orientation frequency" for each inverton as min(R, F)/(R+F) pooled across reads from all samples, we find that 1011/1837 invertons exhibit a minor orientation frequency between 0 and 0.01, 437/1837 invertons exhibit a minor orientation frequency between 0.01 and 0.1, and 389/1837 invertons exhibit a minor orientation frequency between 0.1 and 0.5 (Supplementary Section S1). Note as a caveat that these frequencies depend on bacterial growth condition and should therefore be interpreted cautiously as a rough estimate of flipping tendency in the subset of growth conditions we tested here rather than a characteristic defining parameter inherent to a given inverton.

### Sequence homology in invertible regions enables categorization and motif enrichment analysis of detected invertons

We categorized the 1837 identified invertons into separate inverton groups (Fig. 2A) using homology of their respective IR sequences that flank each inverton. We performed a multiple sequence alignment (MSA) of all 1837 IR sequences, and used the resulting tree of sequences to cluster invertons into groups based on branch length thresholding (Fig. 2B, Supplementary Section S1, Methods–Inverton categorization using IR homology). The optimal branch length threshold was determined using IR sequence motif discoverability as a target metric (Supplementary Section S2, Methods–Inverton categorization using IR homology). This resulted in a total of 146 inverton groups (Fig. 2A), with IR motifs identified for 114/146 inverton groups (Supplementary Table S6). Inverton groups ranged in size from 2 to 72 invertons with a median of 9 invertons per group. Minor orientation frequencies of invertons varied within groups, indicating that invertons with homologous IR sequences could exhibit differing flipping frequencies (Supplementary Section S1), noting also that 133/146 inverton groups had least a single inverton with minor orientation frequency greater than 0.01. Twenty-nine out of the 146 groups had at least 10 invertons from a single phylum, with the largest group (inverton group 131) containing 68/72 invertons from Bacteroidota. Using max inverton count from a single phylum as a metric, we found that the 5 top inverton groups were all dominated by phylum Bacteroidota: after group 131, groups 126, 7, 136, and 143 contained 45/46, 42/46, 37/38, and 35/35 invertons, respectively, from Bacteroidota. We found that the IR motifs from these five groups matched closely to 5 previously published IR motifs found in Bacteroidota by Jiang et al. [14] (Supplementary Section S3). Furthermore, the IR motif for group 138 (which included 32/39 invertons from phylum Verrucomicrobiota, all from *Akkermansia muciniphila* ATCC-BAA-835) closely resembled the previously published IR motif in *Akkermansia muciniphila* [14]. This independent re-discovery using an entirely new metagenomic sequencing dataset of all 6 IR motifs from Jiang et al. validated our inverton detection and grouping approach.

We also report a number of inverton groups with previously unpublished motifs across different phyla. For instance, focusing on the 29 inverton groups with at least 10 invertons from a single phylum, we found novel IR motifs in inverton groups 128, 137, 122, 48, and 68, which all comprised a majority of their invertons from phylum Bacteroidota, or inverton groups 139 and 125 which both comprised a majority of invertons from phylum Firmicutes_A (Supplementary Section S3). While many inverton groups appeared dominated by a single phylum, this was not always the case—as a counterexample, we also observed inverton group 145, which consisted of 20 invertons, 9 of which originated from phylum Bacteroidota (across 5 strains) and 10 of which originated from phylum Firmicutes_A (across 7 distinct strains). MSA of the 20 IR sequences in this group revealed a high degree of conservation even across phyla, and moreover leaves on the IR sequence MSA tree did not neatly cluster by phylum (i.e., IR sequences originating in Bacteroidota were not necessarily more similar to each other than those originating in Firmicutes_A, Supplementary Section S4). These findings suggest that group 145 invertons may have spread across phyla via horizontal gene transfer.

Beyond IR motifs, we next applied motif enrichment search using MEME on full inverton sequences (as opposed to only their IR sequences) to identify motifs enriched in each inverton group (flanking IR sequences were included as part of full inverton sequences, Methods–Motif detection and promoter prediction in invertons). We discovered a total of 266 motifs, spread across 126 inverton groups (Supplementary Table S7). Several identified motifs highly resemble previously reported inverton-associated promoters, consistent with the role of invertons in turning gene expression on/off by changing promoter orientation [14]. For instance, we found motifs highly similar to a previously described Bacteroidota promoter motif [14, 33] independently enriched in 3 distinct Bacteroidota-dominated inverton groups (motifs 126-2, 131-2, and 136-2, Fig. 2C, Supplementary Section S3). Furthermore, we found that invertons in these three groups were differentially distributed between different Bacteroides strains (Supplementary Table S5), including closely related strains. As an example, we found that two strains of *Bacteroides thetaiotaomicron* present in hCom2—VPI-5482 and 1-1-6, ANI estimate 98.8% using fastANI [34]—harbor 15 distinct invertons from groups 126, 131, and 136 (Fig. 2B).

An instance of a motif similar to the described Bacteroidota promoter [14, 33] was found in 14/15 of these invertons (i.e., either motif 126-2, 131-2, or 136-2). Using motif 131-2 as an example, we confirmed that across all of hCom2, instances of this motif tended to be found upstream of and on the same strand as their nearest gene (Fig. 2D), consistent with expectations for a promoter. We observed 566 out of 1217 total motif instances to exhibit this consistent upstream orientation, more than compared to random chance based on 10,000 random samples each of (i) shuffling motif loci—median 32/1217 with consistent upstream orientation, (ii) permuting motif strand—median 417/1217 with consistent upstream orientation, and (iii) permuting gene strand—median 321/1217 with consistent upstream orientation (Fig. 2E). Meanwhile, we also discovered a motif enriched in sequences from inverton group 138 (which consists primarily—32 out of 39—of invertons from *Akkermansia*) that highly resembled the previously described *Akkermansia* promoter motif [14], which also exhibited promoter-like enrichment , though with a lower degree of confidence (Supplementary Section S5).

Beyond testing previously described promoter motifs, we also used random sampling bootstrap to detect new putative promoters. We scanned enriched motifs across all hCom2 metagenomes (Supplementary Table S8), and identified motifs whose detected instances were significantly enriched (*p *< 0.001) on the same strand as and upstream of their nearest gene, meaning observed instances > 9990/10,000 random samples for all three random sampling tests (shuffling motif loci, permuting motif strand, and permuting gene strand). This approach generated a catalog of 8 inverton-associated putative promoter motifs, which included both previously described promoter motifs and several previously undescribed motifs (Supplementary Section S5). Note that based on the *p *< 0.001 cutoff used, the previously described Bacteroidota promoter motif was counted as a putative promoter motif (listed three times independently as motif 126-2, 131-2, and 136-2), but not the previously described *Akkermansia* motif (motif 138-2) [14] (Supplementary Section S5). In addition to putative promoters, we also found motifs enriched on the same strand as and downstream of their nearby gene (Supplementary Section S5), which may be indicative of sequence features associated with transciptional termination or post-transcriptional modification. Collectively, these findings demonstrated the utility of our community-wide bioinformatic search as an approach for motif and promoter discovery.

### Genomic proximity links distinct inverton groups to specific gene families

We next sought to identify gene families that are enriched in regions proximal to (within ±5 kb) or directly intersecting identified invertons. As promoters are known to often be embedded within invertons [14], we further split non-inverton-intersecting genes between "regulatable" genes which could be driven by a promoter in their associated inverton (i.e., 5′-end is proximal to inverton for the given gene and all genes between given gene and inverton), and "non-regulatable" genes that are nevertheless proximal to invertons (Supplementary Table S9).

**Fig. 3:**
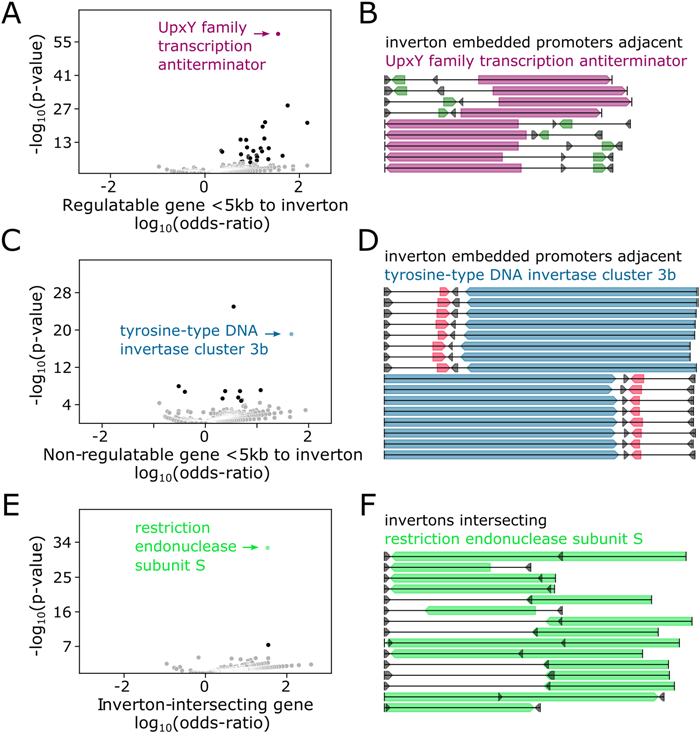
Cell surface products and invertase genes are enriched with consistent orientation near specific inverton groups. **A** Volcano plot of regulatable gene families enriched near invertons based on odds ratio of being observed within 5kb of an inverton, relative to rest of the hCom2 genomes. Gray points are non-significant based on Bonferroni *p* value correction. **B** Genome diagram of all instances of UpxY family transcription antiterminator genes near invertons containing previously described Bacteroides promoter motif [14, 33]—coding sequences annotated with this gene function (marked in purple) are consistently oriented in a way that can be regulated by promoter motif (marked in green) upon inverton flipping. Flanking inverton IR regions marked in black. **C** Volcano plot of non-regulatable gene families enriched near invertons based on odds ratio of being observed within 5 kb of an inverton, relative to rest of the hCom2 genomes. Gray points are non-significant based on Bonferroni *p* value correction. **D** Genome diagram of all instances of tyrosine-type DNA invertase cluster 3b genes near invertons containing previously described Bacteroides promoter motif [14, 33]—coding sequences annotated with this gene function (marked in blue) are consistently oriented in a way that cannot be regulated by promoter motif (marked in red) upon inverton flipping. Flanking inverton IR regions marked in black. **E** Volcano plot of gene families enriched for direct inverton intersection, based on odds ratio of being directly overlapping an inverton, relative to rest of the hCom2 genomes. Gray points are non-significant based on Bonferroni *p* value correction. **F** Genome diagram of all instances of restriction endonuclease subunit S genes near invertons from group number 145. Coding sequences annotated with this gene function (in green) consistently overlap inverton loci. Flanking inverton IR regions marked in black

For each gene family (defined as a unique annotation from the NCBI PGAP pipeline [35]), we counted the number of times genes within (and outside) this family were proximal (or not) to invertons in each of 3 proximity types: "intersecting," "regulatable," or "non-regulatable" orientation. Building 2-by-2 tables of within-vs.-outside and proximal-vs.-not count for each gene, proximity type, and inverton group, we uncovered numerous cases of inverton-group specific gene family enrichment for both "regulatable" and "non-regulatable" genes, as well as genes that directly intersect invertons (Supplementary Table S10). For instance, UpxY family transcription antiterminator family genes significantly enriched (Fisher’s exact *p *< 0.05 with Bonferroni correction) as regulatable genes (Fig. 3A,B) near invertons in Bacteroidota-dominated groups 126, 144, 136, and 137, suggesting possible roles in regulation of exopolysaccharide biosynthesis [9]. Other enriched regulatable genes included many previously described hits related to cell surface products such as TonB-dependent receptor and RagB/SusD family nutrient uptake outer membrane protein (inverton group 131), fimbrillin family protein (inverton groups 137, 112, 122), and PEP-CTERM sorting domain-containing protein (inverton group 138).

Meanwhile, enriched non-regulatable genes included several invertase families such as tyrosine-type DNA invertase cluster 3b (Fig. 3C,D), which was enriched in inverton group 126, as well as tyrosine-type recombinase/integrase (inverton groups 139, 145) and site-specific integrase (inverton groups 136, 126, 137, 48, 116). The presence of cell surface products and invertase genes near invertons aligned with previous reports of similar gene enrichment in gut microbes [14]. Our own results further indicated that invertases near invertons—which are often considered likely candidates for controlling inverton flipping [16]—are generally non-regulatable and thus unlikely to themselves be regulated by inverton-embedded promoters. A potential exception was found in inverton group 145, with tyrosine-type recombinase/integrase gene family found to be enriched in both regulatable as well as non-regulatable orientations near group 145 invertons.

Finally, we also observed instances of gene families enriched for direct intersection with inverton sequences. For instance, invertons from group 145 were significantly enriched with members of the restriction endonuclease subunit S gene family (Fig. 3E,F). This observation aligns with previous reports of restriction enzymes with switchable specificity where recombination and inversion at enzyme coding sequences leads to production of different isoforms of enzyme protein with different specificities [36–39]. In addition to enrichment of restriction endonuclease subunit S and tyrosine-type recombinase/integrase gene families, inverton group 145 was also enriched for the relaxase/mobilization nuclease domain-containing protein and plasmid mobilization relaxosome protein MobC gene families genes, lending further support to the the idea that this group of invertons may have spread via HGT.

### Genomic proximity links distinct inverton groups to specific invertases

**Fig. 4:**
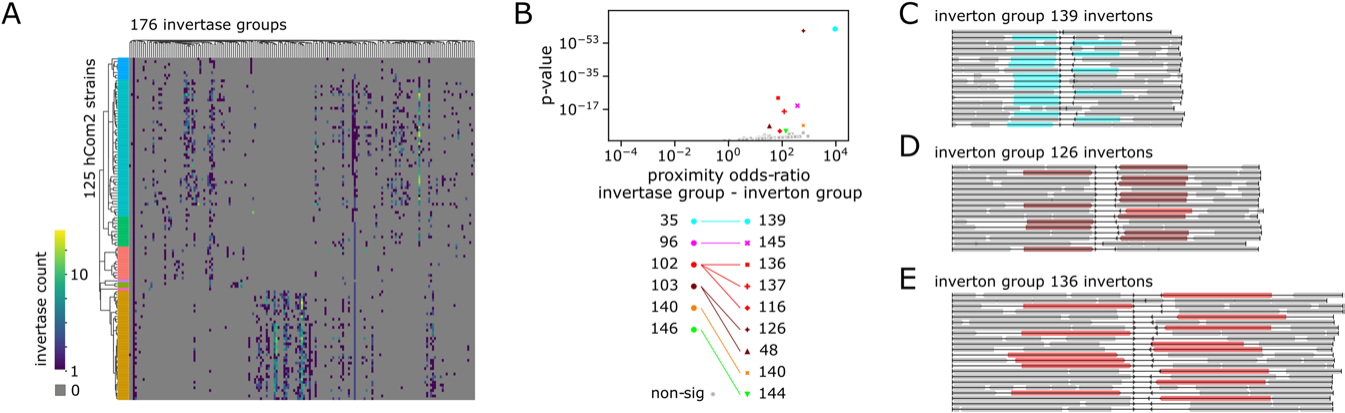
Specific invertase groups are enriched near specific inverton groups. **A** Log-heatmap of invertase counts by hCom2 strain and by invertase group. hCom2 strains organized by phylogeny, phylum colors as in Fig. 1A. **B** Volcano plot of inverton-group to invertase-group pairs, based on odds ratio of invertase in given invertase group being observed within 5 kb of an inverton in given inverton group. Gray points are non-significant based on Bonferroni *p* value correction. Inverton and invertase groups are specified by marker color and shape, respectively, with significant links specified in the legend. **C** Genome diagram of regions surrounding group 139 invertons, with invertase group 35 genes highlighted in blue—for simplification if multiple invertons of this group are found in the same genome, a single representative is plotted. Nearly all invertons in this group have a nearby group 35 invertase. **D** Genome diagram of regions surrounding group 126 invertons, with invertase group 103 genes highlighted in dark red—for simplification if multiple invertons of this group are found in the same genome, a single representative is plotted. **E** Genome diagram of regions surrounding group 136 invertons, with invertase group 102 genes highlighted in light red—for simplification if multiple invertons of this group are found in the same genome, a single representative is plotted

We next explored whether a bioinformatic approach could link identified inverton groups to specific groups of invertase genes, based on the idea that such genes are often located near the invertons they regulate [16]. Gene annotation detected 3932 invertase genes among hCom2 genomes (Supplementary Table S11). We used multiple sequence alignment and clustering to group these invertase genes into 176 invertase groups based on sequence homology (Fig. 4A, Methods–Genome annotation, invertase detection and categorization, Supplementary Section S6). For each of the 126 identified inverton groups, we systematically checked for each of the 176 invertase groups whether the corresponding invertases are enriched in the proximity of the corresponding invertons.

Counting an invertase gene as proximal if it intersects, or is within 5 kb of the inverton, we identified inverton group-invertase group pairs with significantly (Fisher’s exact *p *< 0.05 with Bonferroni correction) enriched proximal invertase counts (Supplementary Table S12). This approach revealed 9 such pairs (Fig. 4B, Methods), representing a total of 135 inverton-invertase examples from 52 different strains including members of both Clostridia and Bacteroidia classes (Supplementary Table S11). The most significant link between such pairs was between inverton group 139—a group of 33 invertons (32 of which are from class Clostridia)—and invertase group 35, a group of genes belonging to the tyrosine-type recombinase/integrase family found in classes Clostridia and Bacteroidia (Fig. 4B,C). Meanwhile, among the Bacteroidota-dominated inverton groups, group 136, 137, and 116 had significant links to invertase group 102, while inverton groups 126 and 48 were instead linked to invertase group 103 (Fig. 4B,D,E). By contrast, other large Bacteroides-dominated inverton groups such as 131 were not significantly linked to any invertase groups (Fig. 4B, Supplementary Section S7). These associations between inverton and invertase groups indicates potential co-evolution of invertase and IR sequences, with instances of multiple inverton groups linked to a single invertase group potentially further suggesting some degree of flexibility in the ability of invertase proteins to control flipping across inverton recognition IR sites.

### Differential analysis between metagenomic sample types indicates inverton-modulated gene expression changes associated with surface adhesion and gut colonization

**Fig. 5:**
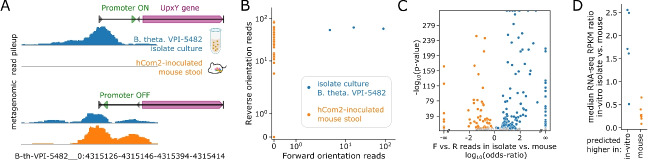
Inverton orientation is modulated by gut colonization and drives differential gene expression. **A** Example of inverton in *Bacteroides thetaiotaomicron* VPI-5482 from inverton group 126 (B-th-VPI-5482__0:4315126-4315146-4315394-4315414) with different forward and reverse read counts depending on growth condition. While roughly equal forward and reverse read support is observed when cultured in vitro, mouse stool derived samples only show reverse orientation read support. **B** Forward vs. reverse read count scatterplot of inverton in **A**, demonstrating that reverse orientation enrichment is observed consistently across all mouse samples. **C** Volcano plot of invertons with directional preference, based on odds ratio of forward vs. reverse reads count for in vitro isolate vs. mouse samples. **D** Median RPKM in vitro vs. mouse ratios for 12 *Bacteroides thetaiotaomicron* VPI-5482 genes predicted to be differentially expressed between in vitro isolate vs. mouse samples based on inverton orientation. Values greater than 1 correspond to in vitro upregulation relative to mouse. Blue and orange dots represent genes predicted to be upregulated in vitro and in mouse, respectively

To investigate how processes of bacterial surface adhesion and gut colonization are linked to inverton orientations at a community-wide level, we compared inverton orientation probabilities between different sample types (Supplementary Table S4). We first identified associations with gut colonization by searching for invertons where our bioinformatic workflow yields a different proportion of sequencing reads supporting the forward and reverse orientations between (i) samples from isolate cultures of individual hCom2 strains and (ii) fecal samples of gnotobiotic mice inoculated with hCom2 (Fig. 5A,B), recovered from mice colonized over a total of 5 generations. As an example, we found an inverton in inverton group 126—B-th-VPI-5482__0:4315126-4315146-4315394-4315414—which had read support for both forward and reverse orientations in *Bacteroides thetaiotaomicron* VPI-5482 isolate culture, but only support for the reverse orientation in mouse-stool sequencing samples (Fig. 5A), a trend that was consistent across 69 mouse stool samples and 3 isolate cultures (Fig. 5B). We identified a total of 224 directionally biased invertons (Fig. 5C) across 53 strains whose forward and reverse read proportions were significantly (Fisher’s exact *p *< 0.05 with Bonferroni correction) different between pooled isolate culture and pooled mouse gut fecal samples (summing forward (F) and reverse (R) orientation read counts pooled across 291 in vitro isolate samples vs. pooled across 69 mouse fecal samples, Methods–Differential analysis of inverton orientation between different microbial growth conditions, Supplementary Table S13). Applying the same approach we next identified associations specifically with surface adhesion by comparing (iii-a) mixed in vitro cultures of hCom2 as a surface attached community using mucin-agar carriers as a synthetic surface and (iii-b) corresponding mucin-agar carrier cultures of hCom2, instead sampled from the liquid-phase. This yielded 38 directionally biased invertons (across 16 strains) whose orientation probabilities were significantly different between mucin-agar surface-attached and liquid-phase samples (Supplementary Table S13).

Cross-referencing these directionally biased invertons against (1) our catalog inverton-embedded putative promoters (Supplementary Section S5, Supplementary Table S8) and (2) locations and orientations of regulatable gene CDSs adjacent to these invertons (Supplementary Table S9), we generated lists of genes whose expression we predicted to be modulated either up or down by inverton-flipping during either gut colonization (comparing isolate culture vs. mouse stool samples) and surface adhesion (comparing carrier-attached vs. supernatant mixed culture samples) (Methods Gene expression prediction, Supplementary Table S14). We found a number of significant (Fisher’s exact *p *< 0.05 with Bonferroni correction) gene families related to cell surface products (Supplementary Table S15). Some, such as the SLBB domain-containing protein and SH3 beta-barrel fold-containing protein, families appeared to be consistently upregulated in mouse stool compared to isolate culture (Supplementary Table S15), suggesting higher expression during gut colonization. Meanwhile, other gene families such as UpxY family transcription antiterminator and polysaccharide biosynthesis/export family protein—both of which are linked to EPS production in Bacteroidota [9]—appeared to be enriched for both down- as well as upregulation (Supplementary Table S15) during gut colonization (i.e., more of these genes are upregulated than would be expected by random chance, and also more of these genes are downregulated than would be expected by random chance), consistent with the notion that cells may be actively remodeling their surface EPS content by turning off production of certain types in favor of others [9]. Comparing carrier-attached vs. supernatant cultures, we found the FimB/Mfa2 family fimbrial subunit gene family was consistently upregulated on carrier attached cultures (Supplementary Table S15), consistent with the known role of these genes in adhesion and biofilm formation [40].

Next, we sought to use publicly available transcriptomic data to validate some of the differential gene expression predictions we made based on directional enrichment of promoter-embedded invertons. We leveraged a recently published RNA-seq dataset of *Bacteroides thetaiotaomicron* VPI-5482 [41], with read libraries derived from both in vitro culture as well as mouse samples (Table S16). Based on our analysis, we computationally predicted a total of 12 differentially expressed genes in *Bacteroides thetaiotaomicron* VPI-5482 when comparing between isolate in vitro culture and growth in mouse, 5 of which we predict to be upregulated in vitro and 7 of which we predict to be upregulated in mouse. To validate these predictions, we calculate the median RNA-seq reads per kilobase per million mapped reads (RPKM) for these 12 genes in both mouse and in vitro RNA-seq data (Table S17). These calculations reveal that the median RPKM in vitro vs. mouse ratios are significantly higher (*p *= 0.0047, two-sided Mann-Whitney *U* test) in genes we predicted as in vitro upregulated than in genes we predicted as mouse upregulated (Fig. 5D), providing preliminary validation for our approach. Collectively, these findings confirmed that surface adhesion and intestinal colonization in complex gut communities directly modulate inverton flipping, and predicted how the expression of key genes are modulated in this process.

### Longitudinal analysis across in vivo and in vitro timepoints

In addition to performing differential analysis between different metagenomic sample types (e.g., isolate culture vs. mouse stool), we also analyzed longitudinal samples across timepoints to better understand inverton dynamics both in vivo and in vitro. For in vivo analysis, we compared samples across mouse generations, and searched for invertons whose forward-vs.-reverse orientations exhibited significant (Fisher’s exact *p *< 0.05 with Bonferroni correction) differences between early and late generation mice (Methods–Longitudinal analysis of inverton orientation across timepoints). We additionally calculate at each timepoint the F.-vs.-R. inversion ratio—defined as reverse over total read counts (R/(R+F)).

**Fig. 6:**
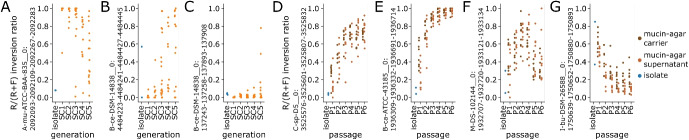
Invertons exhibit dynamic flipping across in vivo mouse generations and in vitro culture passages. **A** Example of inverton in *Akkermansia muciniphila* ATCC-BAA-835 from inverton group 138 (A-mu-ATCC-BAA-835__0:2092093-2092109-2092267-2092283) whose inversion ratio (R/(R+F), so higher scores correspond to more reverse orientation reads) exhibits a dynamic trend across mouse generations, from nearly universal reverse orientation in first generation (SC1) mice, but shifts toward forward orientation in later generations. **B** Example of inverton in *Bacteroides cellulosilyticus* DSM-14838 from inverton group 143 (B-ce-DSM-14838__0:4484223-4484241-4484427-4484445) whose inversion ratio shifts from nearly universal forward orientation in first generation (SC1) mice toward reverse orientation in later generations. **C** Example of inverton in *Bacteroides cellulosilyticus* DSM-14838 from inverton group 131 (B-ce-DSM-14838__0:137243-137258-137893-137908) whose inversion ratio shifts from nearly universal forward orientation in first generation (SC1) mice toward reverse orientation in later generations, with a markedly slower trend than **B**. **D** Example of inverton in *Clostridium sp.* D5 from inverton group 139 (C-sp-D5__0:3525576-3525601-3525807-3525832) whose inversion ratio shifts from mostly forward orientation in passage 1 cultures (P1) toward reverse orientation in later passages. **E** Example of inverton in *Bacteroides caccae* ATCC-43185 from inverton group 131 (B-ca-ATCC-43185__0:1936309-1936332-1936691-1936714) whose inversion ratio shifts rapidly from mostly forward orientation in passage 1 cultures (P1) toward nearly complete reverse orientation by P4/P5. **F** Example of inverton in *Megasphaera* DSMZ-102144 from inverton group 51 (M-DS-102144__0:1932707-1932720-1933121-1933134) whose inversion ratio shifts from mostly forward orientation in passage 1 cultures (P1) toward reverse orientation, before stabilizing at a roughly equal mix of reverse and forward orientation reads. **G** Example of inverton in *Intestinimonas butyriciproducens* DSM-26588 from inverton group 139 (I-bu-DSM-26588__0:1750639-1750652-1750880-1750893) whose inversion ratio shifts from mostly reverse orientation in passage 1 cultures (P1) toward forward orientation in later passages, with consistently higher inversion ratios in mucin-agar carrier cultures (dark brown) than corresponding supernatant cultures (light brown)

Using this approach, we identified a total of 123 invertons (Table S18) across 34 strains that exhibited time-dependent behavior, such as an inverton in *Akkermansia muciniphila* ATCC-BAA-835 from inverton group 138 located near two autotransporter domain containing proteins (A-mu-ATCC-BAA-835__0:2092093-2092109-2092267-2092283). The F.-vs.-R. inversion ratio for this inverton trended from nearly universal reverse orientation in first generation (SC1) mice to majority forward orientation by fifth generation (SC5) mice (Fig. 6A, Supplementary Section S8). Curiously, we also found this type of trend in some—but not all—other group 138 invertons from *Akkermansia muciniphila* (Supplementary Section S9), suggesting the existence of additional layers of regulatory control in determining inverton flipping dynamics beyond simple IR sequence recognition.

Our analysis also revealed a wide range of timescales of inverton dynamics, sometimes even within a single bacterial strain. For instance, within *Bacteroides cellulosilyticus* DSM-14838, we observed two distinct invertons—B-ce-DSM-14838__0:4484223-4484241-4484427-4484445 from inverton group 143 and B-ce-DSM-14838__0:137243-137258-137893-137908 from inverton group 131—that both started from nearly complete forward orientation in SC1 mice and trended toward increasing reverse orientation but with the former doing so at a markedly more rapid rate (Fig. 6B,C). The presence of nutrient uptake genes near the former and exopolysaccharide biosynthesis genes near the latter suggested these two invertons may be responsible for regulating different biological functions (Supplementary Section S8).

Applying this early-vs.-late enrichment approach with mixed in vitro cultures, we also identified invertons whose forward-vs.-reverse orientations were associated with changes across passage timepoints (Table S18), such as an inverton in *Clostridium sp.* D5 from inverton group 139 (C-sp-D5__0:3525576-3525601-3525807-3525832) which exhibits a dynamic shift from mostly forward orientation in passage 1 cultures (P1) toward reverse orientation in later passages (Fig. 6D). As with mouse generational data, we also observed a range of timescales, with an inverton in *Bacteroides caccae* ATCC-43185 from inverton group 131 (B-ca-ATCC-43185__0:1936309-1936332-1936691-1936714) exhibiting particularly a rapid shift from mostly forward orientation in passage 1 cultures (P1) toward nearly complete reverse orientation by P4/P5 (Fig. 6E). Note additionally that timescales involved in in vitro cultures are inherently much shorter than mouse generational data as culture passage intervals are 3 days apart, compared with months between mouse generations.

We also observed invertons with dynamics that do not appear to converge toward fully reverse nor fully forward orientation, such as one in *Megasphaera* DSMZ-102144 from inverton group 51 (M-DS-102144__0:1932707-1932720-1933121-1933134) which exhibits an early shift toward reverse orientation, before stabilizing at a roughly equal mix of reverse and forward orientation reads at later passages (Fig. 6F). Finally, we also observed invertons whose in vitro dynamics appeared to be dependent on surface adhesion, such as inverton in *Intestinimonas butyriciproducens* DSM-26588 from inverton group 139 (I-bu-DSM-26588__0:1750639-1750652-1750880-1750893) which exhibited increasing forward orientation overall, but with consistently higher reverse orientation in surface-attached mucin-agar carrier cultures than corresponding supernatant cultures (Fig. 6G). Carrier-vs.-supernatant dependent dynamics were also observed in a collection of 19 invertons (across 13 different inverton groups) all from the same strain of *Clostridium sp.* D5 that consistently exhibited modest levels of reverse orientation reads in carrier cultures, with virtually no reverse orientation reads in supernatant cultures (Supplementary Section S10). These findings indicate that invertons from different inverton groups (i.e., with disparate IR sequences) can exhibit highly similar trends between surface-attached vs. supernatant cultures over time, while those with highly similar IR sequences can exhibit disparate trends across mouse generations (Supplementary Section S9). Together, these results reinforce the idea that invertons produce a wide range of dynamic behaviors during surface adhesion and gut colonization, and that these dynamics are likely mediated by additional layers of regulatory control beyond IR sequence recognition alone.

### Comparison of different short-read alignment options

To test the robustness of our results to different alignment algorithm options, we repeated our PhaseFinderDC analysis using different bowtie2 settings comparing the –very-sensitive (PhaseFinderDC default option) and –fast presets, as well as a customized setting with increased mismatch and gap open penalties that mimics the –intractg preset (for mapping intra-species contigs to a reference) from the bwa alignment algorithmm [42] (Methods–Inverton detection using defined community sequencing data). We also compared these results with those obtained using the original PhaseFinder algorithm which used bowtie instead of bowtie2. We found that the longitudinal inverton patterns observed in Fig. 6 remained consistent when using different bowtie2 options, although notable differences were detected when comparing against results obtained using the original PhaseFinder algorithm (Supplementary Section S11).

## Methods

### hCom2 mouse generational experiment and metagenomic sequencing

Three male-female pairs of C57/B6 mice (P1) were colonized with hCom2 at age 4–6 weeks of age. These mice were and bred for 4 subsequent generations (F1–F4). Male and female pups were weaned at age 4 weeks and housed in separate cages. Specifically, within each generation, three male-female pairs of mice were selected and bred to produce the subsequent generation. Extra pups that were not used for breeding were housed in separate cages by gender. For each member of each generation, mice were sampled every month for up to 2 years or until the death of the animal. Pups from the parental (P1) generation were labeled as SC1, F1 generation labeled as SC2, F2 generation SC3, F3 generation as SC4, and F4 generation as SC5. At the beginning of each month, fecal sampling was performed and the stool was sequenced. Metagenomic sequencing was performed as previously described [29]: genomic DNA was extracted from pellets using the DNeasy PowerSoil HTP kit (Qiagen) and quantified in 384-well format using the Quant-iT PicoGreen dsDNA Assay Kit (Thermofisher). Sequencing libraries were generated in 384-well format using a custom low-volume protocol based on the Nextera XT process (Illumina). The concentration of DNA from each sample was normalized to 0.18 ng/$\upmu$L using a Mantis liquid handler (Formulatrix). If the concentration was <0.18 ng/$\upmu$L, the sample was not diluted further. Tagmentation, neutralization, and PCR steps of the Nextera XT process were performed on a Mosquito HTS liquid handler (TTP Labtech), leading to a final volume of 4 $\upmu$L per library. During PCR amplification, custom 12-bp dual unique indices were introduced to eliminate barcode switching, a phenomenon that occurs on Illumina sequencing platforms with patterned flow cells. Libraries were pooled at the desired relative molar ratios and cleaned up using Ampure XP beads (Beckman) to achieve buffer removal and library size selection. The cleanup process was used to remove fragments <300 bp or >1.5 kbp. Final library pools were quality-checked for size distribution and concentration using a Fragment Analyzer (Agilent) and qPCR (BioRad). Sequencing reads were generated using a NovaSeq S4 flow cell or a NextSeq High Output kit, in 2 × 150 bp configuration. Five to 10 million paired-end reads were targeted for isolates and 20–30 million paired-end reads for communities.

### Inverton detection using defined community sequencing data

We developed a modified Phasefinder [14] workflow—PhaseFinderDC—to detect invertons from metagenomic read libraries derived from mixtures of hCom2 strains. A custom hCom2 reference database was generated by concatenating microbial genome sequences for all 125 strains in hCom2. As in the original Phasefinder workflow, EMBOSS einverted was then used to locate inverted repeat sequences and thus generate a list of potential invertons. Based on this list, we then created an augmented genomic reference containing both forward and reverse orientation sequences for each potential inverton. This augmented reference then served as the database against which metagenomic sequencing reads are aligned. Here we made a modification to the original Phasefinder workflow for PhaseFinderDC to include all genomic sequences located between potential invertons—in addition to forward/reverse sequences of potential invertons—to aid in filtering of ambiguously aligned reads (discussed further below).

Metagenomic read libraries derived from mouse stool samples were pre-processed using Biobakery kneaddata [43] to remove mouse host DNA reads (median 91.6% of reads passed mouse-host-filtering across 69 mouse samples, Supplementary Table S2); human reads were not explicitly removed. This was skipped for samples derived from pure single-strain and mixed community in vitro cultures. Metagenomic read alignment of read libraries from was then carried out using bowtie2 instead of bowtie (as per original Phasefinder), given that the majority of sequencing reads used in our analyses exceeded 100 bp in length (median 98.8% of reads successfully mapped across 636 analyzed samples, Supplementary Table S2). We set PhaseFinderDC to use the –very-sensitive bowtie2 preset by default (used to generate all presented findings with exception of Supplementary Section S11), with flexibility to run with alternate bowtie2 settings. We use this flexibility to compare how results changed when using the bowtie2 –fast preset as well as a customized setting with increased mismatch and gap open penalties ("–very-sensitive –mp 14,5 –rdg 13,3 –rfg 13,3") that mimics the –intractg from the bwa aligner (Supplementary Section S11).

Using mapping quality (MAPQ) scores reported by bowtie2, PhaseFinderDC filtered out ambiguously aligned reads by removing any alignments with scores below 30. For each potential inverton, counts of read alignments unambiguously supporting forward and reverse orientations based on paired-end orientation (Pe_F, Pe_R) and based on directly spanning inversion junction (Span_F, Span_R) were enumerated as per the original Phasefinder workflow for each sample. For each potential inverton, read counts were pooled across isolate culture samples from the given strain, as well as all hCom2 mouse and in vitro mixed culture samples. We called invertons if forward and reverse orientations are supported by at least 5 read alignments each after pooling, based on both paired-end orientation as well as direct span (i.e., required pooled $Pe\_F>=5, Pe\_R>=5, Span\_F>=5, Span\_R>=5$). This threshold is similar to that suggested by the original PhaseFinder publication [14] which used $Pe\_F>=5, Pe\_R>=5, Span\_F>=3, Span\_R>=3$. The original publication also used a R/(R+F) > 0.01 cutoff, which we omitted here to enable capturing of rarely flipped invertons, compensating instead with a slightly more stringent $Span\_F>=5, Span\_R>=5$ cutoff. This approach enabled us to detect invertons with minor orientation frequency (min(R,F)/(R+F)) between 0 and 0.01, many of which shared similar IR sequence homology with invertons exhibiting higher minor orientation frequencies (Supplementary Section S1). Downstream analysis such as calculation of inversion ratios for each called inverton for each sample used Pe_F, Pe_R counts.

### Inverton categorization using IR homology

IR sequences for all 1837 called invertons were used to generate a multiple sequence alignment using Clustal-omega [44]. Based on this MSA, we used TreeCluster [45] to cluster invertons into distinct groups based on IR sequence homology. We tested a range of tree-distance thresholds (T = 0.01 to 0.99 in increments of 0.01) for generating separate groups, with smaller thresholds generating more (smaller, more closely related) groups of invertons. We then used MEME [46] on IR sequence groups to search for enriched sequence motifs applying -mod zoops -nmotifs 1000 -minw 6 -maxw 100 -objfun classic -revcomp -markov_order 0 -evt 0.05 parameters, counting the total number of groups with a detected motif for each tested tree-distance threshold. We then selected a tree-distance cutoff of T = 0.60 as it maximized the number of groups for which a motif was detected, generating a total of 146 groups of invertons out of which 114 MEME was able to detect an enriched sequence motif in the IR sequences (Supplementary Section S2).

### Motif detection and promoter prediction in invertons

For each of the 146 identified groups of invertons, we used MEME to identify motifs enriched within inverton sequences applying -mod anr -nmotifs 1000 -minw 6 -maxw 100 -objfun classic -revcomp -markov_order 0 -evt 0.05 parameters, now using the full inverton sequences, as opposed to only the IR sequences (flanking IR sequences were also included as part of full inverton sequences). Significantly enriched motifs were then scanned using fimo across all hCom2 genomes, applying a $10^{-8}$ *p* value cutoff to account for the large sequence database size ($4.67\cdot 10^8$ bp total). For each motif instance detected by fimo, we used bedtools closest [47] to identify location and orientation of the closest associated coding sequence. For each motif, we then counted the number of fimo-detected instances that were consistent with those of a promoter, that is to say upstream of its nearest gene, on the same strand. We compared this number against (i) 10,000 random samples where the locations of motif instances were shuffled using bedtools shuffle [47], (ii) 10,000 random samples where the strand orientations of motif instances were permuted, and (iii) 10,000 random samples where the strand orientations of hCom2 genes were permuted. Motifs whose actual count of promoter-consistent instances exceeded 9990/10,000 (>99.9%ile) of all three random samples were identified as putative promoter motifs. Note that as we did not know a priori whether the motif or its reverse complement represented a putative promoter element, we performed these tests for all identified motifs and their reverse complements, reporting the version oriented on the same strand as the nearest gene.

### Gene annotation and enrichment analysis near invertons

All hCom2 genomes were annotated using NCBI PGAP pipeline [35] version 2023-05-17.build6771. We identified enrichment of specific annotations near invertons by counting for each annotation the number of instances (i) gene with given annotation is located within ±5 kb window of any detected inverton, (ii) gene with different annotation is located within ±5 kb window of any detected inverton, (iii) gene with given annotation is not located within ±5 kb window of any detected inverton, and (iv) gene with different annotation is not located within ±5 kb window of any detected inverton. For each gene annotation, we compiled these four counts into a 2 × 2 contingency table, and identified annotations significantly (Fisher’s exact *p *< 0.05 with Bonferroni correction) enriched near invertons. We repeated this analysis independently for each inverton group. We also repeated this analysis by subsetting genes within the ±5 kb window to cases of (a) non-inverton-intersecting genes that were oriented with their 5′ end proximal to the inverton and were either adjacent to the inverton or all intervening genes were also oriented with their 5′ end proximal to the inverton such that a promoter in the inverton could potentially drive expression of said genes—i.e., regulatable, (b) non-inverton-intersecting genes that did not meet the criteria in (a), i.e., non-regulatable, and (c) genes that intersected a detected inverton.

### Invertase detection, categorization, and detection of links to inverton groups

Using PGAP annotations, we focused on extracting all coding sequences whose annotations contained mention of "invertase," "integrase," and "recombinase." Treating these collectively as potential invertase genes, we used Clustal-omega [44] to perform a multiple sequence alignment on the translated amino acid sequences. We used TreeCluster to cluster invertases into distinct groups based on protein homology, using a tree-distance cutoff of 0.8 (Supplementary Section S6) to yield 176 invertase groups. For each invertase group, we counted for each of the 126 inverton groups the number of instances (i) an invertase in the invertase group is located within ±5 kb of an inverton in the inverton group, (ii) an invertase in another invertase group is located within ±5 kb of an inverton in the inverton group, (iii) an invertase in the invertase group is located within ±5 kb of an inverton in another inverton group, and (iv) an invertase in another invertase group is located within ±5 kb of an inverton in another inverton group. For each invertase-group/inverton-group pair, we compiled these four counts into a 2 × 2 contingency table, and identified significantly (Fisher’s exact *p *< 0.05 with Bonferroni correction) linked invertase-group/inverton-group pairs.

### Differential analysis of inverton orientation between different microbial growth conditions

To identify invertons whose orientation significantly differed between different microbial growth conditions A-vs.-B, we compared counts of forward and reverse supporting reads (Pe_F, Pe_R) pooled across all samples from condition A versus condition B. For each inverton, we compiled the four resulting counts (forward reads pooled across condition A, reverse reads pooled across condition A, forward reads pooled across condition B, and reverse reads pooled across condition B) into a 2 × 2 contingency table, and applied a Fisher exact test to identify invertons whose forward-vs.-reverse counts significantly (*p *< 0.05) linked to microbial growth condition, with Bonferroni correction to account for multiple hypothesis testing. We focused on two such A-vs.-B comparisons: (i) samples from pure strain isolate cultures vs. mouse stool samples to explore effect of community colonization in vivo and (ii) samples from in vitro mucin-carrier vs. supernatant cultures.

### Differential gene expression predictions and validation in *Bacteroides thetaiotaomicron*

We predicted genes to be differentially expressed if they were located downstream of an inverton with a putative promoter motif, and the orientation of the associated inverton was also identified as linked to microbial growth conditions. We validated the 12 total such genes that were predicted to be differentially expressed between mouse stool and pure isolate culture in the *Bacteroides thetaiotaomicron* genome by analyzing published RNA-seq dataset from *Bacteroides thetaiotaomicron* that included both pure isolate culture as well as mouse stool samples [41]. We first used hocort [48] to remove mouse transcriptome derived reads from RNA-seq read libraries, then aligned to hCom2 coding sequences using bowtie2 [49]. Read counts were normalized using conditional quantile normalization with the cqn R package [50] to obtain estimates of reads per kilobase per million mapped reads (RPKM). We then calculate ratios of median RPKM between RNA-seq samples from pure isolate culture versus mouse stool samples to validate predicted differential expression.

### Longitudinal analysis of inverton orientation across timepoints

To identify invertons whose orientation significantly differed between early vs. late generation mouse samples, we compared counts of forward and reverse supporting reads (Pe_F, Pe_R) pooled across all samples from mouse generation 1 [SC1] versus mouse generations 2–5 [SC2,SC3,SC4,SC5]. For each inverton, we compiled the four resulting counts (forward reads pooled across generation 1 mice, reverse reads pooled across SC1 mice, forward reads pooled across [SC2,SC3,SC4,SC5] mice, and reverse reads pooled across [SC2,SC3,SC4,SC5] mice) into a 2 × 2 contingency table, and applied a Fisher exact test to identify invertons whose forward-vs.-reverse counts significantly (*p *< 0.05) linked to microbial growth condition, with Bonferroni correction to account for multiple hypothesis testing. We then repeated this analysis with all four possible cutoffs for early vs. late generation ([SC1,SC2]-vs.-[SC3,SC4,SC5], [SC1,SC2,SC3]-vs.-[SC4,SC5], and [SC1,SC2,SC3,SC4]-vs.-[SC5]). Invertons were identified as having significant association with mouse generation timepoint if any of these tests passed the *p *< 0.05 (with Bonferroni correction) threshold. This same approach was used to identify invertons whose orientation significantly differed between early vs. late passage in vitro mixed culture samples (testing ([P1]-vs.-[P2,P3,P4,P5,P6], [P1,P2]-vs.-[P3,P4,P5,P6], [P1,P2,P3]-vs.-[P4,P5,P6], [P1,P2,P3,P4]-vs.-[P5.P6], and [P1,P2,P3,P4,P5]-vs.-[P6]), independently analyzing mucin-carrier-attached and supernatant sample types.

### Data visualization

Custom python and R scripts were developed for data visualization, using the following packages: scipy [51], pandas [52], numpy [53], seaborn [54], python [55], jupyter notebook [56], statsmodels [57], biopython [58], matplotlib [59], R [60], ggtree [61], treeio [62], ggnewscale [63], phytools [64], tidyr [65], dplyr [66], stringr [67], ggplot2 [68], ape [69].

## Discussion

Here we presented a community-wide analysis of invertons in the defined gut microbial community hCom2, providing a customized workflow for detecting invertons in defined microbial communities based on the previously published PhaseFinder algorithm which we used to generate a comprehensive catalog of inverton locations across hCom2. Using the sequence homology found in the IR regions of these detected invertons, we categorized discovered invertons into groups, and used these groups to identify enriched motifs. This uncovered a number of promoter-like motifs—including some which were previously undescribed—whose instances were consistently found upstream of their nearest gene. Analyzing the proximity of invertons to nearby genes, we also revealed links between specific groups of invertons and specific groups of invertases, suggesting a potential regulatory link.

By analyzing large scale metagenomic sequencing of a defined community across multiple sample types, including isolate cultures vs. mouse fecal samples as well as surface-attached vs. liquid phase cultures, we were able to observe differences in inverton orientation probabilities associated with gut colonization and surface adhesion. By detecting directionally biased invertons in more than a third (53/125) of all strains in hCom2, we directly confirmed the hypothesis that colonization and adhesion are associated with inverton dynamics. A key advantage of performing this analysis in hCom2 was the availability of high quality genomes and genome annotations, which enabled us to leverage the discovery of directionally biased invertons into bioinformatic predictions of not only the identity of specific modulated genes, but also the direction of predicted modulation—for instance we predicted that colonization/adhesion are associated with a remodeling of EPS moieties in *Bacteroides*, aligning with previous work [9]. Beyond different sample types, we also identified invertons whose orientations exhibited changes over timepoints both in vivo (across mouse generations) and in vitro (across culture passages). Our findings suggest active inverton dynamics across a range of timescales during surface adhesion and gut colonization, mediated by additional layers of regulatory control beyond IR sequence recognition alone.

We conclude by noting several limitations to our work and point to areas for further exploration. First off, PhaseFinderDC currently uses a highly conservative approach to handle multi-mapping read alignments that arise when closely related strains are included in the same genome database. Namely, reads that are suspected of potential multi-mapping (by default, with a MAPQ score less than 30) are discarded from analysis when enumerating forward and reverse read counts. While this yields a high degree of confidence in the final called invertons, the approach also prevents invertons in regions with high sequence homology from being detected. This problem is amplified when more strain genomes are added to the reference database, as the likelihood of homologous sequences increases with genome database size. For this reason, while we have demonstrated proof-of-principle feasibility with a defined community of 125 strain genomes, PhaseFinderDC is not well suited for use with large generic genome databases such as UHGG [70] or GTDB [32] that contains thousands of strain genomes—a key prerequisite for use in analyzing undefined communities such as stool samples. We envision future work can apply statistical models similar to the approach from Bracken [71] that combine information on the uncertainty level of multi-mapping reads with genome sequencing depth estimates generated from unambiguous reads to make probabilistic read assignments to particular inverted repeat regions. Rather than simply discarding these reads, such an approach would enable detection of invertons in regions with high sequence homology to other strains, and allow PhaseFinderDC to be used with large generic genome databases for analyzing undefined microbial communities like stool.

We note also that as our analyses are computational in nature, our predicted inverton-associated gene expression changes, as well as links between invertase and invertons are only statistical associations. Mechanistic validation of these predictions will require future experimental work, for instance synthetic manipulations such as phase-locked inverton constructs to measure bacterial phenotypes when a particular inverton is locked in a forward or reverse orientation. These synthetic approaches can be combined with more sophisticated readouts such as metatranscriptomics on mixed bacterial communities to validate whether putative promoter motifs identified here indeed drive gene expression as we predict, expanding on the limited example we tested with *Bacteroides thetaiotaomicron* VPI-5482. Beyond promoters, future work should follow-up on additional motifs identified as enriched downstream of CDSs, to investigate whether they play any regulatory roles, for instance as transcriptional terminators. Additional experimental manipulations can also be used to target knockout or over-expression of predicted invertases, to test whether they indeed regulate inverton flipping of their predicted targets.

Furthermore, the current analysis has focused on a limited subset of bacterial growth conditions with minimal environmental stress. Future work could augment the community by incorporating pathogenic taxa, and investigate how more complex environments with the presence of environmental stressors such as antibiotic exposure affect the inverton landscape. By obtaining more sequencing data in variable growth conditions, we would likely also expand the catalog of known invertons, given the observation that invertons flip in certain growth conditions. For instance, while we were only able to call 557 invertons using isolate culture sequencing reads during workflow benchmarking, by augmenting our analysis with sequencing data from different growth conditions (e.g., mixed in vitro culture and mouse stool samples), this increased to 1837 called invertons. Finally, while our work here has focused on inverton-mediated genetic variability, it would be valuable in the future to explore how inverton dynamics across microbial communities are linked to other forms of genetic variability, such as changes that arise as a consequence of mutation and horizontal gene transfer, to more comprehensively profile the cumulative effects of genetic variability on community function.

## Supplementary information

Supplementary Material 1: Supplementary Sections S1-S18 are available in the Supplementary Information PDF file. Supplementary Tables S1-S18 are included as CSV files and contain the following data: Table S1: Metadata on hCom2 strains analyzed in this work, including column information on strain name, abbreviated name (abbrev), associated sequence contigs, BioSample/BioProject accession numbers, and GTDB phylogeny (domain,phylum,class,order,family,genus,species). Table S2: Read libraries analyzed in this work with associated metadata, including column information on sample names, sample types, SRA accession number, sample descriptions, number of read pairs in the raw read library, number and fraction of read pairs that passed mouse-host-filtering (for mouse-stool sample reads only), number and fraction of read pairs that were successfully mapped by bowtie2 (denominator being the number of read pairs that passed mouse-host-filtering if sample was mouse-stool sample, or total raw read pair count otherwise). Table S3: Forward/reverse read counts of invertons in isolate culture comparing original PhaseFinder vs PhaseFinderDC, including information on the actual strain culture used to generated the read library, inverton ID, forward read counts based on paired-end orientation (Pe_F), reverse read counts based on paired-end orientation (Pe_R), forward read counts based on directly spanning inversion junction (Span_F), reverse read counts based on directly spanning inversion junction (Span_R), the strain genome onto which reads were mapped and called as inverton (mappedStrain), which workflow was used (original PhaseFinder of PhaseFinderDC), and whether this was a mis-map (i.e., actualStrain was not the same as mappedStrain). Table S4: Forward/reverse read counts of all invertons across all samples, determined using PhaseFinderDC, including column information on inverton ID, sample ID, forward read counts based on paired-end orientation (Pe_F), reverse read counts based on paired-end orientation (Pe_R), inversion ratio Pe_R/(Pe_R+Pe_F) calculated using read counts based on paired-end orientation (Pe_ratio), forward read counts based on directly spanning inversion junction (Span_F), reverse read counts based on directly spanning inversion junction (Span_R), inversion ratio Span_R/(Span_R+Span_F) calculated using read counts based on paired-end orientation (Span_ratio). Table S5: Metadata on all identified invertons including column information on inverton ID, inverton group, whether it intersects a gene coding sequence (intersectGene), the associated IR sequence, and full inverton sequence. Table S6: Enriched motifs detected across inverton IR sequences, including column information on motif ID and sequence. Table S7: Enriched motifs detected across full inverton sequences, including column information on motif ID and sequence. Table S8: Instances of detected motifs from Table S7 across hCom2 genomes, including column information on genomic locations (chrom, start, stop, strand), the associated motif ID, MEME *p*-value of the detected instance. For motif instances that intersect an inverton, additional column information is included on the inverton ID, location of the inverton (start_inverton, end_inverton), and inverton group. Table S9: Metadata on inverton-proximal genes across hCom2, including column information on genomic loci of each gene (chrom, start, end, strand), the gene ID, the gene annotation, the associated inverton ID, and whether the gene intersected the inverton directly, and if not whether it could be regulated by a promoter in the inverton (5’-end of gene is closer to inverton than 3’-end of gene). Table S10: Enrichment of inverton-proximal gene annotations by inverton group and type of proximity, including column information on the gene annotation, inverton group, the type of proximity (regulatable-vs.-nonregulatable-vs.-intersecting), the number of genes with given annotation that are proximal to inverton of given group with given type of proximity (near inverton gene count), the number of genes with given annotation that are not proximal to inverton of given group with given type of proximity (not near inverton gene count), the number of genes not with given annotation that are proximal to inverton of given group with given type of proximity (near inverton other gene count), the number of genes not with given annotation that are not proximal to inverton of given group with given type of proximity (not near inverton other gene count), the odds ratio, Fisher’s Exact *p*-value, and significance after multiple hypothesis correction. Table S11: Invertase genes across hCom2, including column information on the locations of the genes (chrom, start, end, strand), gene ID, gene annotation, and invertase group. For invertase gene instances that intersect an inverton, additional column information is included on the inverton ID and inverton group. Table S12: Enrichment of invertase groups proximal to inverton groups, including column information on the inverton group, invertase group, the number of invertons within given inverton group that are proximal to an invertase gene within the given invertase group, the number of invertons not within given inverton group that are proximal to an invertase gene within the given invertase group, the number of invertons within given inverton group that are not proximal to an invertase gene within the given invertase group, the number of invertons not within given inverton group that are not proximal to an invertase gene within the given invertase group, the odds ratio, Fisher’s Exact *p*-value, and significance after multiple hypothesis correction. Table S13: Directionally biased invertons comparing between (i) isolate culture / mouse stool and (ii) carrier-attached / liquid-phase mixed culture samples, including column information on inverton ID, the number of forward orientation reads in the first condition (Pe_F_cond1), the number of reverse orientation reads in the first condition (Pe_R_cond1), the number of forward orientation reads in the second condition (Pe_F_cond2), the number of reverse orientation reads in the second condition (Pe_R_cond2), the odds ratio, Fisher’s Exact *p*-value, and label of first condition vs. second condition comparison. Table S14: Gene regulation predictions based on directionally biased invertons with promoter motifs, including column information on the genomic location and attributes of the gene (chrom, start, end, gene ID, gene strand, gene annotation), the inverton ID, the type of gene proximity (regulatable-vs.-nonregulatable-vs.-intersecting), the number of forward inverton orientation reads in the first condition (Pe_F_cond1), the number of reverse inverton orientation reads in the first condition (Pe_R_cond1), the number of forward inverton orientation reads in the second condition (Pe_F_cond2), the number of reverse inverton orientation reads in the second condition (Pe_R_cond2), the odds ratio, Fisher’s Exact *p*-value, the label of first condition vs. second condition comparison, the direction the inverton is enriched toward in the first condition, the motif ID of the predicted promoter, the strand of the detected motif on the genome, whether the promoter is a reverse complement of the motif, the strand of the promoter determined based on prior 2 columns, and the condition with the predicted upregulation. Table S15: Gene annotations enriched for inverton-based regulation, including column information on the gene annotation, the label of first condition vs. second condition comparison, the condition with the predicted upregulation, the number of genes with given annotation that are predicted to be upregulated in the given condition, the number of genes with a different annotation that are predicted to be upregulated in the given condition, the number of genes with given annotation that are not predicted to be upregulated in the given condition, the number of genes with a different annotation that are not predicted to be upregulated in the given condition, the odds ratio, and Fisher’s Exact *p*-value. Table S16: Metadata on transcriptomic datasets used for gene regulation prediction validation, including column information on the SRA accession, gene ID, sample type, and cqn-normalized RPKM value. Table S17: Gene expression estimated based on transcriptomic datasets, including column information on the gene ID, median RPKM estimated across mouse and in vitro RNA-seq samples, and the predicted upregulated condition based on inverton orientation data. Table S18: Directionally biased invertons comparing across timepoints (mouse generation and mixed culture passage), including column information on the inverton ID, the number of forward inverton orientation reads in the first condition (Pe_F_cond1), the number of reverse inverton orientation reads in the first condition (Pe_R_cond1), the number of forward inverton orientation reads in the second condition (Pe_F_cond2), the number of reverse inverton orientation reads in the second condition (Pe_R_cond2), the odds ratio, Fisher’s Exact *p*-value, the label of first condition vs. second condition comparison.

## Data Availability

The mouse stool sequencing data generated in this study have been deposited in the NCBI database under BioProject accession code PRJNA1119053. In vitro cultures were analyzed using previously published data [29, 30], as well as new sequencing data that have been deposited in the NCBI database under BioProject accession code PRJNA1119029. Metadata for all metagenomic read libraries analyzed can be found in Supplementary Table S2. Key processed data generated in this study are provided in the Supplementary Tables. PhaseFinderDC code available at: https://github.com/xiaofanjin/PhaseFinderDC. Code and additional data used for analysis and visualization available at: https://github.com/xiaofanjin/hcom2-invertons.

## References

[1] Zarrinpar A, Chaix A, Yooseph S, Panda S. Diet and feeding pattern affect the diurnal dynamics of the gut microbiome. Cell Metab. 2014;20(6):1006–17. 25470548 10.1016/j.cmet.2014.11.008 PMC4255146

[2] Bäckhed F, Roswall J, Peng Y, Feng Q, Jia H, Kovatcheva-Datchary P, et al. Dynamics and stabilization of the human gut microbiome during the first year of life. Cell Host Microbe. 2015;17(5):690–703. 25974306 10.1016/j.chom.2015.04.004

[3] Halfvarson J, Brislawn CJ, Lamendella R, Vázquez-Baeza Y, Walters WA, Bramer LM, et al. Dynamics of the human gut microbiome in inflammatory bowel disease. Nat Microbiol. 2017;2(5):1–7. 10.1038/nmicrobiol.2017.4 PMC5319707 28191884

[4] Garud NR, Good BH, Hallatschek O, Pollard KS. Evolutionary dynamics of bacteria in the gut microbiome within and across hosts. PLoS Biol. 2019;17(1):e3000102. 30673701 10.1371/journal.pbio.3000102 PMC6361464

[5] Tropini C, Earle KA, Huang KC, Sonnenburg JL. The gut microbiome: connecting spatial organization to function. Cell Host Microbe. 2017;21(4):433–42. 10.1016/j.chom.2017.03.010. 28407481 10.1016/j.chom.2017.03.010 PMC5576359

[6] McCallum G, Tropini C. The gut microbiota and its biogeography. Nat Rev Microbiol. 2024;22(2):105–18. 37740073 10.1038/s41579-023-00969-0

[7] Earle KA, Billings G, Sigal M, Lichtman JS, Hansson GC, Elias JE, et al. Quantitative imaging of gut microbiota spatial organization. Cell Host Microbe. 2015;18(4):478–88. 26439864 10.1016/j.chom.2015.09.002 PMC4628835

[8] Abraham JM, Freitag CS, Clements JR, Eisenstein BI. An invertible element of DNA controls phase variation of type 1 fimbriae of Escherichia coli. Proc Natl Acad Sci. 1985;82(17):5724–7. 2863818 10.1073/pnas.82.17.5724 PMC390624

[9] Krinos CM, Coyne MJ, Weinacht KG, Tzianabos AO, Kasper DL, Comstock LE. Extensive surface diversity of a commensal microorganism by multiple DNA inversions. Nature. 2001;414(6863):555–8. 11734857 10.1038/35107092

[10] Coyne MJ, Weinacht KG, Krinos CM, Comstock LE. Mpi recombinase globally modulates the surface architecture of a human commensal bacterium. Proc Natl Acad Sci. 2003;100(18):10446–51. 12915735 10.1073/pnas.1832655100 PMC193581

[11] Coyne MJ, Comstock LE. Niche-specific features of the intestinal bacteroidales. J Bacteriol. 2008;190(2):736–42. 17993536 10.1128/JB.01559-07 PMC2223690

[12] Gauntlett JC, Nilsson HO, Fulurija A, Marshall BJ, Benghezal M. Phase-variable restriction/modification systems are required for Helicobacter pylori colonization. Gut Pathog. 2014;6:1–5. 25349630 10.1186/s13099-014-0035-z PMC4209511

[13] Porter NT, Canales P, Peterson DA, Martens EC. A subset of polysaccharide capsules in the human symbiont Bacteroides thetaiotaomicron promote increased competitive fitness in the mouse gut. Cell Host Microbe. 2017;22(4):494–506. 28966055 10.1016/j.chom.2017.08.020 PMC5830307

[14] Jiang X, Hall AB, Arthur TD, Plichta DR, Covington CT, Poyet M, et al. Invertible promoters mediate bacterial phase variation, antibiotic resistance, and host adaptation in the gut. Science. 2019;363(6423):181–7. 30630933 10.1126/science.aau5238 PMC6543533

[15] Yan W, Hall AB, Jiang X. Bacteroidales species in the human gut are a reservoir of antibiotic resistance genes regulated by invertible promoters. npj Biofilms Microbiomes. 2022;8(1):1. 35013297 10.1038/s41522-021-00260-1 PMC8748976

[16] Trzilova D, Tamayo R. Site-specific recombination-how simple DNA inversions produce complex phenotypic heterogeneity in bacterial populations. Trends Genet. 2021;37(1):59–72. 33008627 10.1016/j.tig.2020.09.004 PMC7755746

[17] Van Der Woude MW, Baumler AJ. Phase and antigenic variation in bacteria. Clin Microbiol Rev. 2004;17(3):581–611. 15258095 10.1128/CMR.17.3.581-611.2004 PMC452554

[18] Chanin RB, West PT, Wirbel J, Gill MO, Green GZ, Park RM, et al. Intragenic DNA inversions expand bacterial coding capacity. Nature. 2024;634:234–42. 10.1038/s41586-024-07970-4 39322669

[19] Milman O, Yelin I, Kishony R. Systematic identification of gene-altering programmed inversions across the bacterial domain. Nucleic Acids Res. 2023;51(2):553–73. 36617974 10.1093/nar/gkac1166 PMC9881135

[20] Beachey EH. Bacterial adherence: adhesin-receptor interactions mediating the attachment of bacteria to mucosal surfaces. J Infect Dis. 1981;143(3):325–45. 7014727 10.1093/infdis/143.3.325

[21] Testerman TL, McGee DJ, Mobley HL. Adherence and colonization. Helicobacter pylori Physiol Genet. 2001;379–417.

[22] Chagnot C, Zorgani MA, Astruc T, Desvaux M. Proteinaceous determinants of surface colonization in bacteria: bacterial adhesion and biofilm formation from a protein secretion perspective. Front Microbiol. 2013;4:303. 24133488 10.3389/fmicb.2013.00303 PMC3796261

[23] Lee SM, Donaldson GP, Mikulski Z, Boyajian S, Ley K, Mazmanian SK. Bacterial colonization factors control specificity and stability of the gut microbiota. Nature. 2013;501(7467):426–9. 23955152 10.1038/nature12447 PMC3893107

[24] Sicard JF, Le Bihan G, Vogeleer P, Jacques M, Harel J. Interactions of intestinal bacteria with components of the intestinal mucus. Front Cell Infect Microbiol. 2017;7:387. 28929087 10.3389/fcimb.2017.00387 PMC5591952

[25] Nishiyama K, Yokoi T, Sugiyama M, Osawa R, Mukai T, Okada N. Roles of the cell surface architecture of Bacteroides and Bifidobacterium in the gut colonization. Front Microbiol. 2021;12:754819. 34721360 10.3389/fmicb.2021.754819 PMC8551831

[26] Round JL, Lee SM, Li J, Tran G, Jabri B, Chatila TA, et al. The Toll-like receptor 2 pathway establishes colonization by a commensal of the human microbiota. Science. 2011;332(6032):974–7. 21512004 10.1126/science.1206095 PMC3164325

[27] Wen J, Zhang H, Chu D, Chen X, Feng J, Wang Y, Liu G, Zhang Y, Li Y, Ning K. Deep learning revealed the distribution and evolution patterns for invertible promoters across bacterial lineages. Nucleic Acids Res. 2024;52(21):12817–30. 10.1093/nar/gkae966 PMC11602134 39460615

[28] Zhao C, Shi ZJ, Pollard KS. Pitfalls of genotyping microbial communities with rapidly growing genome collections. Cell Syst. 2023;14(2):160–76. 36657438 10.1016/j.cels.2022.12.007 PMC9957970

[29] Cheng AG, Ho PY, Aranda-Díaz A, Jain S, Yu FB, Meng X, et al. Design, construction, and in vivo augmentation of a complex gut microbiome. Cell. 2022. 10.1016/j.cell.2022.08.003. 10.1016/j.cell.2022.08.003 PMC9691261 36070752

[30] Jin X, Yu FB, Yan J, Weakley AM, Dubinkina V, Meng X, et al. Culturing of a complex gut microbial community in mucin-hydrogel carriers reveals strain-and gene-associated spatial organization. Nat Commun. 2023;14(1):3510. 37316519 10.1038/s41467-023-39121-0 PMC10267222

[31] Langmead B, Trapnell C, Pop M, Salzberg SL. Ultrafast and memory-efficient alignment of short DNA sequences to the human genome. Genome Biol. 2009;10:1–10. 10.1186/gb-2009-10-3-r25 PMC2690996 19261174

[32] Parks DH, Chuvochina M, Rinke C, Mussig AJ, Chaumeil PA, Hugenholtz P. GTDB: an ongoing census of bacterial and archaeal diversity through a phylogenetically consistent, rank normalized and complete genome-based taxonomy. Nucleic Acids Res. 2022;50(D1):D785–94. 34520557 10.1093/nar/gkab776 PMC8728215

[33] Bayley DP, Rocha ER, Smith CJ. Analysis of cepA and other Bacteroides fragilis genes reveals a unique promoter structure. FEMS Microbiol Lett. 2000;193(1):149–54. 11094294 10.1111/j.1574-6968.2000.tb09417.x

[34] Jain C, Rodriguez-R LM, Phillippy AM, Konstantinidis KT, Aluru S. High throughput ANI analysis of 90K prokaryotic genomes reveals clear species boundaries. Nature Commun. 2018;9(1):5114. 30504855 10.1038/s41467-018-07641-9 PMC6269478

[35] Tatusova T, DiCuccio M, Badretdin A, Chetvernin V, Nawrocki EP, Zaslavsky L, et al. NCBI prokaryotic genome annotation pipeline. Nucleic Acids Res. 2016;44(14):6614–24. 27342282 10.1093/nar/gkw569 PMC5001611

[36] Dybvig K, Yu H. Regulation of a restriction and modification system via DNA inversion in Mycoplasma pulmonis. Mol Microbiol. 1994;12(4):547–60. 7934878 10.1111/j.1365-2958.1994.tb01041.x

[37] Dybvig K, Sitaraman R, French CT. A family of phase-variable restriction enzymes with differing specificities generated by high-frequency gene rearrangements. Proc Natl Acad Sci. 1998;95(23):13923–8. 9811902 10.1073/pnas.95.23.13923 PMC24968

[38] De Ste Croix M, Vacca I, Kwun MJ, Ralph JD, Bentley SD, Haigh R, et al. Phase-variable methylation and epigenetic regulation by type I restriction–modification systems. FEMS Microbiol Rev. 2017;41(Supp_1):S3–S15. 10.1093/femsre/fux025 28830092

[39] Atack JM, Guo C, Litfin T, Yang L, Blackall PJ, Zhou Y, et al. Systematic analysis of REBASE identifies numerous type I restriction-modification systems with duplicated, distinct hsdS specificity genes that can switch system specificity by recombination. Msystems. 2020;5(4):10–1128. 10.1128/mSystems.00497-20 PMC7394358 32723795

[40] Park Y, Simionato MR, Sekiya K, Murakami Y, James D, Chen W, et al. Short fimbriae of Porphyromonas gingivalis and their role in coadhesion with Streptococcus gordonii. Infect Immun. 2005;73(7):3983–9. 15972485 10.1128/IAI.73.7.3983-3989.2005 PMC1168573

[41] Kennedy MS, Zhang M, DeLeon O, Bissell J, Trigodet F, Lolans K, et al. Dynamic genetic adaptation of Bacteroides thetaiotaomicron during murine gut colonization. Cell Rep. 2023;42(8):113009. 10.1016/j.celrep.2023.113009 PMC10528517 37598339

[42] Li H, Durbin R. Fast and accurate short read alignment with Burrows-Wheeler transform. Bioinformatics. 2009;25(14):1754–60. 19451168 10.1093/bioinformatics/btp324 PMC2705234

[43] Beghini F, McIver LJ, Blanco-Míguez A, Dubois L, Asnicar F, Maharjan S, et al. Integrating taxonomic, functional, and strain-level profiling of diverse microbial communities with bioBakery 3. Elife. 2021;10:e65088. 33944776 10.7554/eLife.65088 PMC8096432

[44] Larkin MA, Blackshields G, Brown NP, Chenna R, McGettigan PA, McWilliam H, et al. Clustal W and Clustal X version 2.0. Bioinformatics. 2007;23(21):2947–8. 17846036 10.1093/bioinformatics/btm404

[45] Balaban M, Moshiri N, Mai U, Jia X, Mirarab S. TreeCluster: clustering biological sequences using phylogenetic trees. PLoS ONE. 2019;14(8):e0221068. 31437182 10.1371/journal.pone.0221068 PMC6705769

[46] Bailey TL, Johnson J, Grant CE, Noble WS. The MEME suite. Nucleic Acids Res. 2015;43(W1):W39–49. 25953851 10.1093/nar/gkv416 PMC4489269

[47] Quinlan AR, Hall IM. BEDTools: a flexible suite of utilities for comparing genomic features. Bioinformatics. 2010;26(6):841–2. 20110278 10.1093/bioinformatics/btq033 PMC2832824

[48] Rumbavicius I, Rounge TB, Rognes T. HoCoRT: host contamination removal tool. BMC Bioinformatics. 2023;24(1):371. 37784008 10.1186/s12859-023-05492-w PMC10544359

[49] Langmead B, Salzberg SL. Fast gapped-read alignment with Bowtie 2. Nat Methods. 2012;9(4):357–9. 22388286 10.1038/nmeth.1923 PMC3322381

[50] Hansen KD, Irizarry RA, Wu Z. Removing technical variability in RNA-seq data using conditional quantile normalization. Biostatistics. 2012;13(2):204–16. 22285995 10.1093/biostatistics/kxr054 PMC3297825

[51] Virtanen P, Gommers R, Oliphant TE, Haberland M, Reddy T, Cournapeau D, et al. SciPy 1.0: fundamental algorithms for scientific computing in Python. Nat Methods. 2020;17:261–72. 10.1038/s41592-019-0686-2. 32015543 10.1038/s41592-019-0686-2 PMC7056644

[52] pandas development team T. pandas-dev/pandas: Pandas. Zenodo. 2020. 10.5281/zenodo.3509134.

[53] Harris CR, Millman KJ, van der Walt SJ, Gommers R, Virtanen P, Cournapeau D, et al. Array programming with NumPy. Nature. 2020;585(7825):357–62. 10.1038/s41586-020-2649-2. 32939066 10.1038/s41586-020-2649-2 PMC7759461

[54] Waskom ML. seaborn: statistical data visualization. J Open Source Softw. 2021;6(60):3021.

[55] Van Rossum G, Drake Jr FL. Python reference manual. Amsterdam: Centrum voor Wiskunde en Informatica Amsterdam; 1995.

[56] Kluyver T, Ragan-Kelley B, Pérez F, Granger B, Bussonnier M, Frederic J, et al. Jupyter Notebooks – a publishing format for reproducible computational workflows. In: Loizides F, Schmidt B, editors. Positioning and power in academic publishing: players, agents and agendas. Amsterdam: IOS Press; 2016. p. 87–90.

[57] Seabold S, Perktold J. statsmodels: econometric and statistical modeling with Python. In: 9th Python in Science Conference. Austin: Scipy; 2010.

[58] Cock PJA, Antao T, Chang JT, Chapman BA, Cox CJ, Dalke A, et al. Biopython: freely available Python tools for computational molecular biology and bioinformatics. Bioinformatics. 2009;25(11):1422–3. 19304878 10.1093/bioinformatics/btp163 PMC2682512

[59] Hunter JD. Matplotlib: a 2D graphics environment. Comput Sci Eng. 2007;9(3):90–5. 10.1109/MCSE.2007.55.

[60] R Core Team. R: a language and environment for statistical computing. Vienna, Austria; 2019. https://www.r-project.org/. Accessed 19 Feb 2024.

[61] Yu G, Smith DK, Zhu H, Guan Y, Lam TTY. ggtree: an r package for visualization and annotation of phylogenetic trees with their covariates and other associated data. Methods Ecol Evol. 2017;8(1):28–36. 10.1111/2041-210X.12628.

[62] Wang LG, Lam TTY, Xu S, Dai Z, Zhou L, Feng T, et al. Treeio: an R package for phylogenetic tree input and output with richly annotated and associated data. Mol Biol Evol. 2020;37(2):599–603. 10.1093/molbev/msz240. 31633786 10.1093/molbev/msz240 PMC6993851

[63] Campitelli E. ggnewscale: multiple fill and colour scales in ‘ggplot2’. 2022. https://cran.r-project.org/package=ggnewscale. Accessed 19 Feb 2024.

[64] Revell LJ. phytools: an R package for phylogenetic comparative biology (and other things). Methods Ecol Evol. 2012;3(2):217–23. 10.1111/j.2041-210X.2011.00169.x.

[65] Wickham H, Girlich M. tidyr: tidy messy data. 2022. https://cran.r-project.org/package=tidyr. Accessed 19 Feb 2024.

[66] Wickham H, François R, Henry L, Müller K. dplyr: a grammar of data manipulation. 2021. https://cran.r-project.org/package=dplyr. Accessed 19 Feb 2024.

[67] Wickham H. stringr: simple, consistent wrappers for common string operations. 2019. https://cran.r-project.org/package=stringr. Accessed 19 Feb 2024.

[68] Wickham H. ggplot2: elegant graphics for data analysis. Springer-Verlag New York; 2016. https://ggplot2.tidyverse.org. Accessed 19 Feb 2024.

[69] Paradis E, Schliep K. ape 5.0: an environment for modern phylogenetics and evolutionary analyses in R. Bioinformatics. 2019;35:526–8. 30016406 10.1093/bioinformatics/bty633

[70] Almeida A, Nayfach S, Boland M, Strozzi F, Beracochea M, Shi ZJ, et al. A unified catalog of 204,938 reference genomes from the human gut microbiome. Nat Biotechnol. 2021;39(1):105–14. 10.1038/s41587-020-0603-3. 32690973 10.1038/s41587-020-0603-3 PMC7801254

[71] Lu J, Breitwieser FP, Thielen P, Salzberg SL. Bracken: estimating species abundance in metagenomics data. PeerJ Comput Sci. 2017;3:e104. 10.7717/peerj-cs.104 PMC12016282 40271438

